# Influence of Stress Urinary Incontinence and Pelvic Organ Prolapse on Depression, Anxiety, and Insomnia—A Comparative Observational Study

**DOI:** 10.3390/jcm13010185

**Published:** 2023-12-28

**Authors:** Urszula Kalata, Andrzej Pomian, Michał Jarkiewicz, Vitalii Kondratskyi, Krzysztof Lippki, Ewa Barcz

**Affiliations:** 1 Chair of Gynecology and Obstetrics, Medical Faculty, Collegium Medicum, Cardinal Stefan Wyszynski University, 01-938 Warsaw, Poland; u.kalata@uksw.edu.pl (U.K.); a.pomian@uksw.edu.pl (A.P.); vitali.kondracki@gmail.com (V.K.); krzysztof.lippki@gmail.com (K.L.); 2 3rd Department of Psychiatry, Institute of Psychiatry and Neurology, 02-957 Warsaw, Poland; mjarkiewicz@ipin.edu.pl

**Keywords:** urinary incontinence, pelvic organ prolapse, depression, anxiety, insomnia

## Abstract

Background: Among pelvic floor disorders (PFDs), overactive bladder is a well-recognized condition affecting mental health. The aim of this study was to assess whether there is a correlation between stress urinary incontinence (SUI), pelvic organ prolapse (POP), and mental health in comparison to control subjects and whether objective or subjective aspects of diseases are responsible for the aforementioned symptoms. Methods: 192 patients with SUI, 271 with symptomatic prolapse (>2 in the POPQ scale), and 199 controls without pelvic floor disorders were included in this study. Patients completed questionnaires assessing levels of depression, anxiety, and insomnia. The 1-h pad test and IIQ-7 questionnaires were collected in SUI. The pelvic organ prolapse quantification scale and the POPDI6, UDI6, and CRADI-8 questionnaires were used in POP patients. Results: Higher scores in psychiatric scales were observed in SUI (*p* < 0.05) and POP (*p* < 0.05) compared to control. There were no correlations between the objective severity of PFDs and psychological symptoms, while subjective complaints correlated with psychological health. In conclusion, we showed that subjective perceptions of SUI and POP are factors that augment psychiatric symptoms, while objective severity is not correlated with mental status. Conclusions: Our findings suggest that patients with PFDs necessitate multidisciplinary attention, including psychiatric care.

## 1. Introduction

Over 15% of women worldwide suffer from pelvic floor disorders (PFDs) [1]. Stress urinary incontinence (SUI), overactive bladder syndrome (OABs), and pelvic organ prolapse (POP) are the most prevalent urogynecological issues that adversely affect the health and wellbeing of patients [2]. Pelvic floor disorders cause numerous symptoms such as urinary urgency, leakage on exertion, voiding disfunction, hesitancy during urination, post voiding leakage. POP, apart from the physical feeling of bulge, often causes urination problems in the form of difficult initiation of micturition, a feeling of incomplete emptying of the bladder, postmicturition retention, and urinary tract infections. Additionally, difficult defecation, constipation, incomplete emptying of the rectum, loss of gases and stools may occur [3]. All these symptoms may be accompanied by sexual dysfunction caused by pain, lack of self-acceptance, shame, and lack of trust in the partner or his acceptance [4]. All the abovementioned symptoms lead to very serious health, social, professional, and personal consequences. Patients with PFDs tend to give up professional activity, isolate themselves socially, and limit sexual contacts. They often feel socially and personally excluded. All this can potentially lead to deterioration in quality of life in many aspects, lack of self-acceptance, and thus deterioration of mood, anxiety, depression, and sleep disorders. It was demonstrated that PFDs may have a negative influence on different aspects of mental health. Overactive bladder syndrome is the most recognized condition, which harms sleep quality and psychological health. The significant negative impact of these symptoms was described by several authors, cited below, indicating its importance for mental health. Jacomo et al. demonstrated that more than 50% of patients with OAB exhibit symptoms of depression and the strongest factor in its development is previous gynecological surgery [5]. In Melotti’s study [6], 274 women with confirmed diagnosis of OAB underwent a questionnaire survey. The authors showed that depressive symptoms were present in 59.8% of patients and anxiety was diagnosed in 62.4%. Moreover, there was a correlation between the intensity of OAB symptoms with depression and anxiety. The symptoms were mainly related to urgency incontinence and nocturia. A population-based Taiwanese study found a significantly higher risk of depression, anxiety, dementia, and psychotic symptoms in OAB patients [7].

The negative contribution of urgency, pollakisuria, and nocturia on mental health was proved by demonstrating that successful solifenacin treatment leads to a lower BDI (Beck depression inventory) score after 3 months of pharmacotherapy [8]. All these observations indicate the importance of analyzing other PFDs that may affect mental health.

The coexistence of stress urinary incontinence and pelvic organ prolapse with depression, anxiety, and sleep disorders is less recognized. It has been suggested that the severity of postpartum depression may be influenced by SUI [9]. The authors attempted to identify risk factors related to both conditions and demonstrated possible associations between them. A European multicenter study found that 20–40% of women with urinary incontinence assessed the negative impact of this condition on their quality of life, including the occurrence of depression and anxiety [10]. In a cross-sectional study in Lithuania, conducted on 80 patients, a higher incidence of depression and anxiety disorders was found in patients with SUI than in the healthy group; however, it should be noted that the study was conducted on a small group of patients, and it was only a survey study, without objective verification of the problem. Symptoms of depression and anxiety were most pronounced in patients with massive loss of urine and obese patients [11].

Norwegian studies conducted in an older population showed that depression and anxiety are correlated with SUI but to a lower extent as compared with mixed urinary incontinence and OABs [12].

The connection between mental health and POP is even less described. There is a lack of consensus regarding the extent to which prolapse-related symptoms have an impact on depression, anxiety, or sleep disorders. In a Korean study of patients with rectal prolapse, the relationship between depression and the patient’s quality of life was confirmed; however, the authors did not compare psychiatric symptoms with patients without PFDs [13]. The relationship between POP and mental symptoms seemed to be indirectly confirmed by the observations of Ai and colleagues, who showed a reduction in the intensity of these complaints in patients who received pessary therapy due to pelvic organ prolapse [14]. Some authors have also indicated that successful treatment of pelvic organ prolapse allows for the return to quality of life from before the disease, including the emotional state. This suggests that organ prolapse itself is one of the causes of anxiety or depressive disorders [15].

The analysis of the cause-and-effect relationship between the pelvic floor and psychiatric disorders seems to be of great importance. Care for a patient with PFDs is primarily aimed at improving quality of life. This may be achieved using causal treatment of urinary incontinence or pelvic organ prolapse; however, in the case of cooccurrence of mental disorders, actions should be taken to eliminate them at the same time. Therefore, it is important to answer the question of whether depression, anxiety, and sleep disorders in patients with PFDs are primary and unrelated to the disease, or whether treatment of PFDs is itself able to improve mental health.

Even though the current literature provides some data connecting PFDs with mental health, there is a lack of analysis of which aspect of PFDs influences depression, anxiety, and sleep quality in the most pronounced way. Therefore, the current study aimed to find out if there are differences between patients with SUI or POP and control subjects with no PFDs in depression, anxiety, and insomnia scales. Since PFDs adversely affect the quality of life of patients, the deterioration in this respect is not always directly proportional to the objective severity of the problem. A second endpoint was to determine whether objective severity or subjective self-recognized symptoms are responsible for mental health deterioration.

## 2. Materials and Methods

This study was conducted between the years 2021 and 2023 in a tertiary urogynecological center. Patients enrolled in this study were divided into 3 groups:

1. The control group consisted of patients without PFDs who attended the clinic for colposcopy, PAP smear, or ultrasound examination with no pathologies found both where PFDs and other gynecological problems were concerned.
2. SUI group: patients with bothering SUI (everyday leakage, daily use of absorbent pads, positive caught test).
3. POP group: patients with symptomatic prolapse (POPQ > 2) (feeling of bulge, urination or defecation disorders, urgency, impaired sexual activity).

To eliminate potential errors in the interpretation of the results, patients who had concurrent SUI and POP were excluded from this study.

Epidemiological data were collected (age, parity, BMI, comorbidities, and duration of PFDs symptoms).

After obtaining written consent, all patients underwent an urogynecological examination by three highly experienced gynecologists. In the SUI arm, additional results from 1-h pad tests (according to IUGA/ICS) and IIQ7 questionnaires (Incontinence Impact Questionnaire—short form) were collected. In IIQ7, all seven domains were assessed (ability to perform household chores, physical recreation, entertaining activities, ability to travel, participation in social activities, emotional health, and feeling frustrated). In patients with POP, stage of prolapse in the POPQ scale was evaluated; PFDI 20 (Pelvic Floor Distress Inventory) consisting of POPDI6 (Pelvic Organ Prolapse Distress Inventory), UDI 6 (Urogenital Distress Inventory), and CRADI-8 (Colo-Rectal Distress Inventory), as well as PFIQ (Pelvic Floor Impact Questionnaire) questionnaires, were filled in.

All patients included in this study completed questionnaires evaluating depression (CESD—Centre for Epidemiologic Studies Depression Scale), anxiety (STAI—State-Trait Anxiety Inventory), and insomnia (ISI—Insomnia Severity Index).

This study was approved by the local Medical Ethical Committee (KB/1264).

Descriptive statistical analysis expressing the quantitative and categorical variables was performed with the use of R version 4.3.0 software (R Core Team, Vienna, Austria). Normality was tested using the Lillefors and Shapiro–Wilk tests. We associated the degree and type of nonadherence using the U Mann–Whitney test. The Pearson correlation test was used to determine the correlation between quantitative variables. *p*-value < 0.05 was considered statistically significant.

## 3. Results

Six hundred and twenty-two patients were enrolled in this study.

The control group consisted of 199 patients without PFDs; the SUI group: 192 patients; and the POP group: 271 patients. Demographic data are shown in Table 1.

The mean value of the 1-hour pad test was 54 ± 63 g per hour in the SUI group.

The most frequently diagnosed pelvic organ prolapse was stage III in the anterior and apical compartment (63%), stage III in the apical compartment (17%), stage III in the posterior compartment (12%), and stage IV (8%).

Comparison between the control group (*n* = 199) and patients suffering from SUI (*n* = 192) showed that the incidence of SUI causes higher scores in CESD (depression): 30.4 ± 9.6 vs. 32.8 ± 10.2, *p* < 0.02; a higher score in ISI (anxiety): 81.1 ± 13.5 vs. 86.9 ± 16.0, *p* < 0.001; as well as higher scores in ISI (insomnia): 7.6 ± 6.0 vs. 10.9 ± 5.9, *p* < 0.001.

Similarly, we showed, in bothersome POP patients (*n* = 271), higher scores in CESD (depression), 30.4 ± 9.6 vs. 32.1 ± 9.2, *p* = 0.06; STAI (anxiety): 81.1 ± 13.5 vs. 84.2 ± 16.6, *p* = 0.03; and ISI (insomnia): 7.6 ± 6.0 vs. 9.7 ± 5.9, *p* < 0.001 as compared with subjects without PFDs. We did not find differences in STAI, CESD, and ISI scales between pre- and postmenopausal patients in any of the groups (*p* < 0.1)

We also tried to find correlations between the severity of PFDs and mental status. We considered both objective and subjective symptoms’ severity.

We showed that, in the case of incontinent patients, there was no correlation between the objective severity of the leakage shown in a 1-h pad test and mental health in all examined fields (r = −0.12 STAI; r = 0.06 ISI and r = −0.02 in CESD questionnaire).

**Figure 1:**
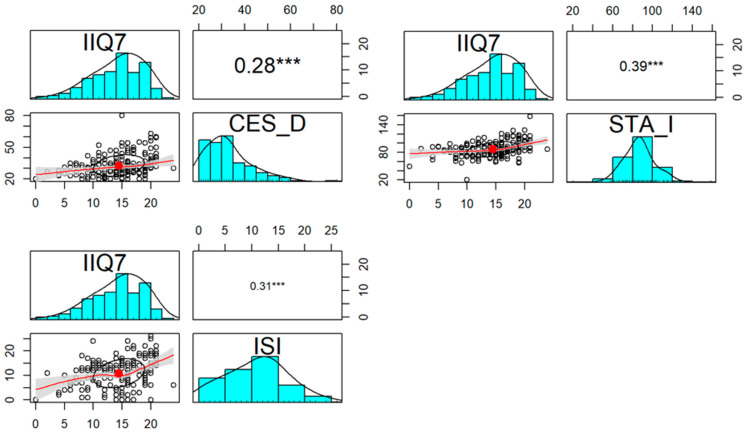
Correlation of CES-D, STAI, and ISI questionnaires and IIQ7 in SUI group. Distribution, scatterplot with 0.95 confidence area (gray shade), correlation ellipse shape (red dot surrounded by a black circle) and correlation coefficient with the significance level (***—*p* < 0.001) for each pair are marked, respectively.

At the same time, we showed a significant correlation between scores in all psychiatric questionnaires and subjective self-esteem questionnaire IIQ7 (Figure 1).

Among specific complaints analyzed in IIQ7 in SUI patients, those that influenced their mental status most heavily were inability to maintain ability to do household chores, entertaining activities, ability to travel, participation in social activities, emotional health, and feeling frustrated. Lower physical activity was the only one that was not connected with psychological questionnaires (Table 2).

We did not confirm the correlation between the POP stage (objective POP severity) shown in the POPQ scale and anxiety, depression, and insomnia (*p* = 0.12).

**Figure 2:**
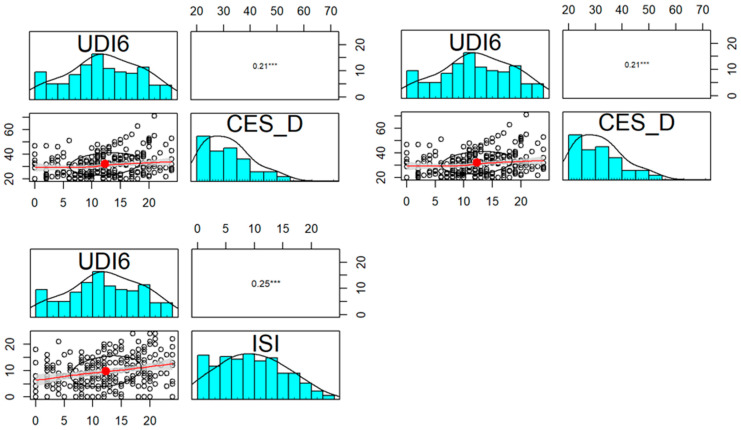
Correlation of CES-D, STAI, and ISI questionnaires and UDI-6 in POP group. Distribution, scatterplot with 0.5 confidence area (gray shade), correlation ellipse shape (red dot surrounded by a black circle), and correlation coefficient with the significance level (***—*p* < 0.001) for each pair are marked, respectively.

**Figure 3:**
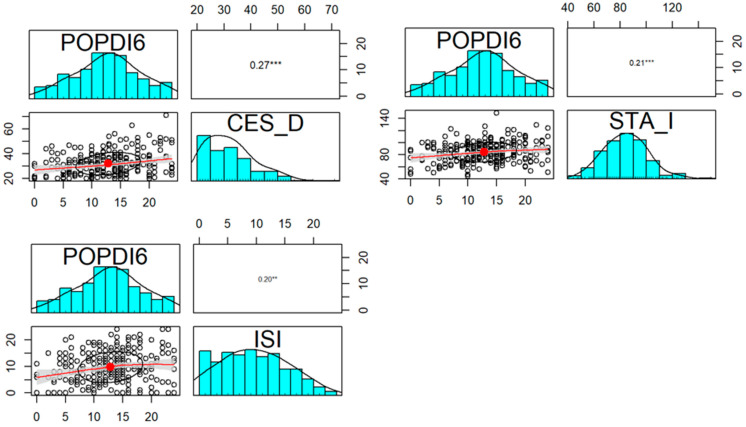
Correlation of CES-D, STAI, and ISI questionnaires and POPDI-6 in POP group. Distribution, scatterplot with 0.5 confidence area (gray shade), correlation ellipse shape (red dot surrounded by a black circle), and correlation coefficient with the significance level (***—*p* < 0.001; **—*p* < 0.01) for each pair are marked, respectively.

**Figure 4:**
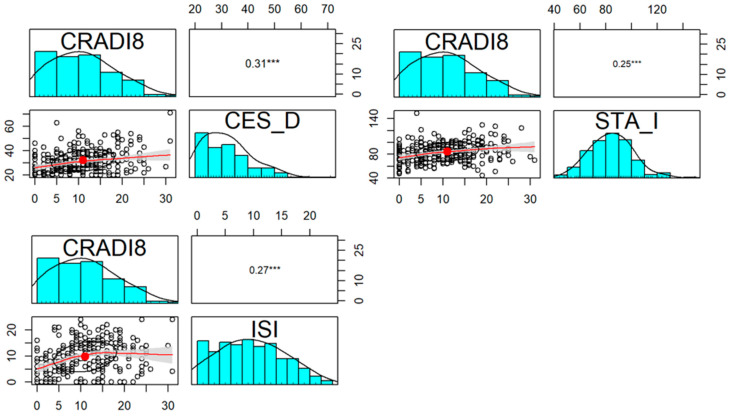
Correlation of CES-D, STAI, and ISI questionnaires and CRADI-8 in POP group. Distribution, scatterplot with 0.5 confidence area (gray shade), correlation ellipse shape (red dot surrounded by a black circle), and correlation coefficient with the significance level (***—*p* < 0.001) for each pair are marked, respectively.

However, similarly to incontinent patients, our observations revealed a correlation between the psychiatric status and the severity of subjective complaints of the examined women as far as lower urinary tract UDI-6 (Figure 2), prolapse symptoms POPDI-6 (Figure 3), and colorectal CRADI-8 (Figure 4) are concerned.

Among specific complaints connected with the pelvic organ prolapse reported by the POP group, pressure in the lower abdomen was significantly connected with all of the psychiatric complaints (anxiety, insomnia, and depression), heaviness in the pelvic area correlated with both anxiety and depression, bulge falling out the vagina with anxiety, uncomplete voiding with insomnia and depression, and necessity to push up the bulge in the vagina with depression (Table 3).

In the case of urogenital distress inquiry symptoms occurring in POP patients, pain and discomfort in the lower abdominal or genital area correlated with all psychiatric scales, as leakage related to physical activity with insomnia and depression (Table 4)**.**

Specific colorectal complaints in POP patients were analyzed: straining during bowel movements, feeling of incomplete bowel emptying, losing gas, urgency during bowel movements all correlated with psychiatric problems (Table 5).

## 4. Discussion

Pelvic floor disorders are a growing health problem in the world. The aging of society and the burden of obesity are the primary causes of this phenomenon. The growing awareness of health and the desire for a better quality life in women all over the world has led to a greater frequency of reporting the issue to health care providers and a better understanding of PFD epidemiology [11]. PFDs cause various symptoms and complaints, including lower urinary tract, colorectal, and vaginal symptoms, as well as sexual impairment [3,16]. PFDs may have a negative impact on social functioning, professional activity, and family relations, as well as causing social stigmatization [17]. Despite the very troublesome symptoms associated with pelvic floor diseases, there are very large differences in how often patients seek help. The level of healthcare-seeking behavior ranges from only 21.3% in Pakistan to 73.4% in USA. It is noteworthy that even in highly developed countries, one-fourth of patients hide their ailments. This depends on the health awareness of patients and is lower in countries with a low degree of economic development [18]. This phenomenon may further perpetuate the feeling of abandonment and social exclusion and thus lead to mood disorders and anxiety.

Whether and how PFDs influence mental health and what the most important factors causing mental health disorders are have been widely discussed [19,20]. Whether PFDs themselves deteriorate psychological status or whether mental problems cause a worse perception of physical problems have also been debated and considered. Regardless of the cause-and-effect relationship, it seems to be important to provide patients with pelvic floor diseases with care focused on their emotional state. This has been demonstrated in relation to overactive bladder syndrome in particular [21].

An overactive bladder has been shown to have a negative impact on depression, sleep disorders, and anxiety in many studies [22] but there is not much research linking stress-related urinary incontinence and pelvic organ prolapse with mental health problems. In the present study, we demonstrated that both SUI and POP negatively influence anxiety, symptoms associated with depression, and increased insomnia compared to subjects with no PFDs in a large group of women. The effect of stress urinary incontinence on anxiety and depression was demonstrated in the Zuluaga COBaLT study; however, the authors noted the high prevalence of mental health issues in patients with SUI without comparing them to healthy individuals. What seems extremely important in the cited study is the fact that both depression and anxiety occurred much more often in women than in men. The abovementioned results remain in agreement with our observations and confirm that it is not only the urgency that deteriorates mental health but also the bothersome episodes of stress urinary leakage [23].

In another observational study, the authors did not show a difference between various types of urinary incontinence and the incidence of depression and anxiety in the Hospital Depression and Anxiety Scale (HADS) [24]; however, both types of incontinence had a negative impact on the examined field. Even though the study was conducted on a small group of patients (73) and consisted only of the questionnaire assessment of symptoms, it stays in agreement with our results showing the negative influence of SUI on mental health. However, the patients qualified for their observation were much younger than in our population (mean age 38.3 ± 3 years), which makes both observations difficult to compare objectively.

In patients with pelvic organ prolapse, we observed a higher prevalence of depression, anxiety, and insomnia in comparison to those who did not have POP. There are not many studies linking POP with mental health status, and it is therefore difficult to compare our results to those obtained by others.

In a recent study, Pizzarro-Berdichevsky et al. indicated that depression in conjunction with pelvic organ prolapse can lead to a worsening of symptoms in PFDI-20 (pelvic floor distress inquiry) [25]. Because the severity of depression was not related to the objective degree of POP, the authors concluded that depression causes aggravation of symptoms in patients. However, it should be noted that the subjective experience of prolapse symptoms is a multifactorial phenomenon. This is influenced by the level of education, health awareness, and expected quality of life.

It has been demonstrated that approximately 30% of postmenopausal women with POP experience depression, and this condition is primarily associated with lower urinary tract symptoms and bowel dysfunction [26]. The same group of authors suggested that there is a similar dependence between POP and anxiety [27]. The studies of the authors cited above are closest to our results, although they were conducted on a much smaller group of patients. In the above publications, as in our observations, it was the subjective severity of symptoms that had the greatest impact on the occurrence of psychiatric disorders in the study groups. What is also important here is the fact that the studies of the cited authors were conducted on postmenopausal patients. In our study, over 73% of patients were in menopause; however, menopausal status did not affect the tested parameters.

We showed that psychological symptoms became worse in patients with stress urinary incontinence and prolapse. Nonetheless, our study fails to provide a definitive answer to the inquiry regarding whether PFDs are responsible for mental disorders or whether mental health has a detrimental impact on the self-recognition of PFDs symptoms. Additionally, it cannot be ruled out that the patient’s upcoming surgery and other personal disturbances could have an additional negative impact on their psychiatric symptoms.

Therefore, further investigations assessing the impact of successful SUI and POP treatment on examined parameters are required. It seems that the most valuable observation in this respect would be to conduct such tests in patients before and after PFD treatment, i.e., after the symptoms have subsided.

Moreover, it seems important to be aware of whether the severity of SUI is the main cause of anxiety, depression, and insomnia, or if other factors matter more. The same questions arise regarding pelvic organ prolapse. Is there a correlation between the objective POPQ measurements and the severity of depression, anxiety, or insomnia, or are there other coexisting ailments such as voiding difficulties, urgency frequency syndrome, and other similar conditions that may have a more significant impact on mental health?

We did not find a correlation between the objective severity of SUI, shown as 1-h pad test results, and STAI, ISI, and CES-D scores in affected patients. Simultaneously, we observed a correlation between the examined domains, namely, depression, anxiety, and insomnia, and the subjective assessment of the quality of life to urinary incontinence in the IIQ7 questionnaire. Our results are the only ones in the literature that show this phenomenon; however, similar results have been published for overactive bladder syndrome, anxiety, and depression [28].

Furthermore, following the findings of incontinent patients, we demonstrated that the objective severity of prolapse as measured by the POPQ scale is not the sole factor influencing life quality, but rather the deterioration of life quality in various aspects, including lower urinary tract, colorectal, and prolapse symptoms, which contribute to the occurrence of anxiety, depression, and insomnia symptoms.

In Drages’ study, it was shown that the subjective severity of prolapse, determined in the International Consultation on Incontinence Questionnaire—Vaginal Symptoms score, is connected with greater psychological distress [29]. Authors who focused on rectal prolapse also found a connection between subjective complaints in the CRADI 8 questionnaire and depressive symptoms [13]. The aforementioned outcomes stay in agreement with the current outcomes. However, we further demonstrated that the objective severity does not influence mental health. Similar observations were made by authors dealing with fecal incontinence. They concluded that depressive disorders related to fecal incontinence are more affected by the severity of the subjective symptoms or the surrounding environment than the objective indicators derived from the test [30].

The study by Piers and colleagues showed that the severity of SUI among nurses and midwives, in a group of over 5000 respondents, showed that the subjective (assessed by women) severity of urinary incontinence on exertion is one of the elements that encourage patients to resign from work [31]. This is once again confirmed by the fact that the subjective experience of symptoms affects the quality of life, mood disorders of patients, as well as life decisions, including decisions about further professional life.

## 5. Conclusions

Both stress urinary incontinence and pelvic organ prolapse are connected to depression, anxiety, and insomnia. Subjective perceptions of SUI and POP are factors that augment those symptoms, while objective severity is not correlated with mental status. The treatment of SUI and POP should then be tailored to each patient’s specific signs and conditions, including mental health issues, to achieve both physiological and psychological success. The obtained findings also suggest that patients with PFDs necessitate multidisciplinary medical attention, including psychological and psychiatric care. Further studies are needed to explain whether successful treatment of PFDs resolves anxiety, depression, and sleep disorders.

## Figures and Tables

**Table 1:** Demographic data of enrolled population.

	Control Group (*n* = 191)	SUI Group (*n* = 192)	POP Group (*n* = 271)
Age (years)	48.5 ± 14	53.3 ± 11.3 ns	60.9 ± 11.7 *p* < 0.05
BMI	29.3 ± 8.4	28.5 ± 9.3 ns	29.1 ± 9.4 ns
Parity (no of deliveries)	1.89 ± 2.3	1.89 ± 1.3 ns	1.96 ± 2.8 ns
Mean duration of symptoms (years)		4.31 ± 4.12 ns	5.34 ± 3.21 ns
Diabetes mellitus type II (%)	9.1	11.1	12.2
Hypertension (%)	7.5	9.0	8.7
Postmenopausal status (%)	54	61 ns	67 *p* < 0.05

**Table 2:** Correlation (r) between domains in the IIQ7 questionnaire and anxiety (STAI), insomnia (ISI), and depression (CESD) scales.

	IIQ7_1	IIQ7_2	IIQ7_3	IIQ7_4	IIQ7_5	IIQ7_6	IIQ7_7	IIQ7
STA_I	0.228 *p* < 0.05	0.115 ns	0.196 *p* < 0.05	0.197 *p* < 0.05	0.276 *p* < 0.05	0.463 *p* < 0.05	0.363 *p* < 0.05	0.383 *p* < 0.05
ISI	0.339 *p* < 0.05	0.125 ns	0.198 *p* < 0.05	0.254 *p* < 0.05	0.173 *p* < 0.05	0.252 *p* < 0.05	0.178 *p* < 0.05	0.309 *p* < 0.05
CES_D	0.2357 *p* < 0.05	0.119 ns	0.240 *p* < 0.05	0.137 *p* < 0.05	0.199 *p* < 0.05	0.314 *p* < 0.05	0.223 *p* < 0.05	0.307 *p* < 0.05

IIQ7_1: ability to perform household chores; IIQ7_2: physical activity; IIQ7_3: entertaining activity; IIQ7_4: ability to travel; IIQ7_5: participation in social activities; IIQ7_6: emotional health; IIQ7-7: feeling frustrated.

**Table 3:** Correlation (r) between POP complaints and psychiatric scales.

	POPDI_1	POPDI_2	POPDI_3	POPDI_4	POPDI_5	POPDI_6
STA_I	0.245 *p* < 0.05	0.321 *p* < 0.05	0.150 *p* < 0.05	0.072 ns	0.12 ns1	0.082 ns
ISI	0.194 *p* < 0.05	0.094 Ns	0.087 ns	0.060 ns	0.221 *p* < 0.05	0.147 ns
CES_D	0.274 *p* < 0.05	0.235 *p* < 0.05	0.050 ns	0.123 ns	0.159 *p* < 0.05	0.236 *p* < 0.05

POPDI_1: pressure in the lower abdomen; POPDI_2: heaviness in the pelvic area; POPDI_3: bulge falling out the vagina; POPDI_4: uncomplicate bowel emptying; POPDI_5: uncomplete voiding; POPDI_6: necessity to push up bulge in the vagina.

**Table 4:** Correlation (r) between UDI-specific complaints and anxiety, insomnia, and depression scales.

	UDI_1	UDI_2	UDI_3	UDI_4	UDI_5	UDI_6
STA_I	0.022 ns	0.009 ns	0.122 ns	0.138 *p* < 0.05	0.020 ns	0.217 *p* < 0.5
ISI	0.118 ns	0.027 ns	0.190 *p* < 0.05	0.134 ns	0.131 ns	0.287 *p* < 0.05
CES_D	0.038 ns	0.060 ns	0.180 *p* < 0.05	0.131 ns	0.083 ns	0.221 *p* < 0.05

UDI_1: frequent urination; UDI_2: leakage connected with urge feeling; UDI_3: urine leakage connected to physical activity; UDI_4: small amounts of urine leakage; UDI_5: difficulty in emptying bladder; UDI _6: pain and discomfort in the lower abdominal area.

**Table 5:** Correlation (r) between specific colorectal complaints in POP patients and anxiety, insomnia, and depression scales.

	CRADI_1	CRADI_2	CRADI_3	CRADI_4	CRADI_5	CRADI_6	CRADI_7	CRADI_8
STA_I	0.140 *p* < 0.05	0.189 *p* < 0.05	0.094 ns	0.163 *p* < 0.05	0.242 *p* < 0.05	0.128 ns	0.194 *p* < 0.05	0.101 ns
ISI	0.210 *p* < 0.05	0.176 *p* < 0.05	0.138 *p* < 0.05	0.103 ns	0.158 *p* < 0.05	0.164 *p* < 0.05	0.208 *p* < 0.05	0.164 *p* < 0.05
CES_D	0.182 *p* < 0.05	0.168 *p* < 0.05	0.149 *p* < 0.05	0.124 ns	0.185 *p* < 0.05	0.184 *p* < 0.05	0.172 *p* < 0.05	0.127 ns

CRADI_1: Feel you need to strain too hard to have a bowel movement? CRADI_2: Feel you have not completely emptied your bowels and the end of bowel movements? CRADI_3: Usually lose stool beyond your control if stool is well formed? CRADI_4: Usually lose your stool beyond your control if the stool is loose? CRADI_5: Usually lose gas from the rectum beyond your control? CRADI_6: Usually have pain when you pass your stool? CRADI_7: Experience a strong sense of urgency and have to rush to the bathroom to have a bowel movement? CRADI_8: Does part of your bowel pass through the rectum and bulge outside during bowel movement?

## Data Availability

Data are stored in medical records in the clinic, unavailable due to privacy and RODO restrictions.
